# The heterogeneous pharmacological medical biochemical network PharMeBINet

**DOI:** 10.1038/s41597-022-01510-3

**Published:** 2022-07-11

**Authors:** Cassandra Königs, Marcel Friedrichs, Theresa Dietrich

**Affiliations:** Bielefeld University, Bioinformatics/Medical Informatics Department, Bielefeld, 33615 Germany

**Keywords:** Data integration, Databases

## Abstract

Heterogeneous biomedical pharmacological databases are important for multiple fields in bioinformatics. Hetionet is a freely available database combining diverse entities and relationships from 29 public resources. Therefore, it is used as the basis for this project. 19 additional pharmacological medical and biological databases such as CTD, DrugBank, and ClinVar are parsed and integrated into Neo4j. Afterwards, the information is merged into the Hetionet structure. Different mapping methods are used such as external identification systems or name mapping. The resulting open-source Neo4j database PharMeBINet has 2,869,407 different nodes with 66 labels and 15,883,653 relationships with 208 edge types. It is a heterogeneous database containing interconnected information on ADRs, diseases, drugs, genes, gene variations, proteins, and more. Relationships between these entities represent drug-drug interactions or drug-causes-ADR relations, to name a few. It has much potential for developing further data analyses including machine learning applications. A web application for accessing the database is free to use for everyone and available at https://pharmebi.net. Additionally, the database is deposited on Zenodo at 10.5281/zenodo.6578218.

## Abstract (summary)

**Table:** 

Measurement(s)	data integration objective
Technology Type(s)	database creation objective

## Background & Summary

In the field of bioinformatics, there are a lot of different heterogeneous biomedical pharmacological databases like Pharmacogenomics Knowledgebase (PharmGKB)^1^, DrugBank^2^ and Comparative Toxicogenomics Database (CTD)^3^. Each database has its own specific field: DrugBank is e.g. a database that focuses on bioinformatics and cheminformatics which mainly contains data about drugs and drug targets^2^. In contrast, PharmGKB is a data source that focuses on drug responses considering variations in the human genome^1^. Heterogeneous data are used for novel biological or medical discoveries, education, or improving diagnostic processes^4,5^. Multiple applications^5–9^ use various heterogeneous biomedical and pharmacologic databases to analyse research questions. For example, Hetionet^8^ is a heterogeneous biomedical Neo4j database that was used for drug repurposing by using meta path prediction. Many approaches use similar databases like DrugBank, Gene Ontology (GO)^10,11^ or PharmGKB^5,7,8^.

Adverse drug reactions (ADRs) represent a field in which a lot of research has been done so far utilizing multiple databases^12–15^. A reason for this is the increasing number of medication errors^16,17^ and ADRs^18^ over the last decades. In Germany for older people, the number of ADR increased from 1,615 (2000) to 5,357 (2016) which shows a higher increase than for younger people^19^. ADRs are the unexpected reaction of the human body caused by pharmacotherapy^12^. In the worst case, ADRs can be the cause of a patient’s death^20^. Approximately 3.8% of all hospital admissions in Europe are caused by ADRs, and 10.1% of all patients suffer from ADRs during their hospitalization^21^. Moreover, a two-year Sicilian study shows that around 6.2% of hospital admissions are related to ADRs^22^. Also, researchers from the UK and US suggest 5–10% of ADR incidences for patients^12^. Additionally, it was shown that genetic variants can result in different reactions to the same pharmacotherapy^23^. The exact relation between ADRs and their underlying molecular mechanisms of genes, proteins, and gene variants is often unknown. There are multiple reasons for an ADR^24^. Some of the reasons which cause ADRs are drug-drug interactions and dosage errors^25,26^. Summed up, a lot of different factors need to be considered for the analysis of ADRs. The necessity for a database of heterogeneous information on drugs, ADRs, genes, proteins, gene variants, and diseases is given.

Many different researchers have collected information about ADR, drugs, and molecular information. One is CTD which contains information about chemical-gene-disease relationships and also considers relationships to phenotypes^3^. CTD provides no information about drug-drug interactions or genetic variants. GraphSAW^27^ is another system with heterogeneous data which is focused on drug interactions and side effects (SEs), equally used in this text to ADR. However, it is not freely available due to the use of licensed data sources and does not consider genetic variants. Hetionet contains information about SEs, compounds, and genes, see Fig. 3. The problem is that the number of SEs is limited and only drugs for 136 diseases are considered^8^. Additionally, it has no drug-drug interaction or genetic variant information. More databases with ADR information are DrugBank^2^, ClinVar^28^, PharmGKB^1^ and ADReCS-Target^23^. Each of these databases is missing some information, for example, drug-drug interaction or gene variation.

Combining different data sources is always challenging and leads to multiple problems. First, each data source provides its own heterogeneous formats ranging from plain text formats to whole Structured Query Language (SQL) databases^29^. Second, the connection between different data source entities is difficult. Mostly, each data source has its own identifier and different kind of information^30^. Mapping between each data source is so far challenging as different entities require individually implemented mapping strategies^29,31^.

**Fig. 1:**
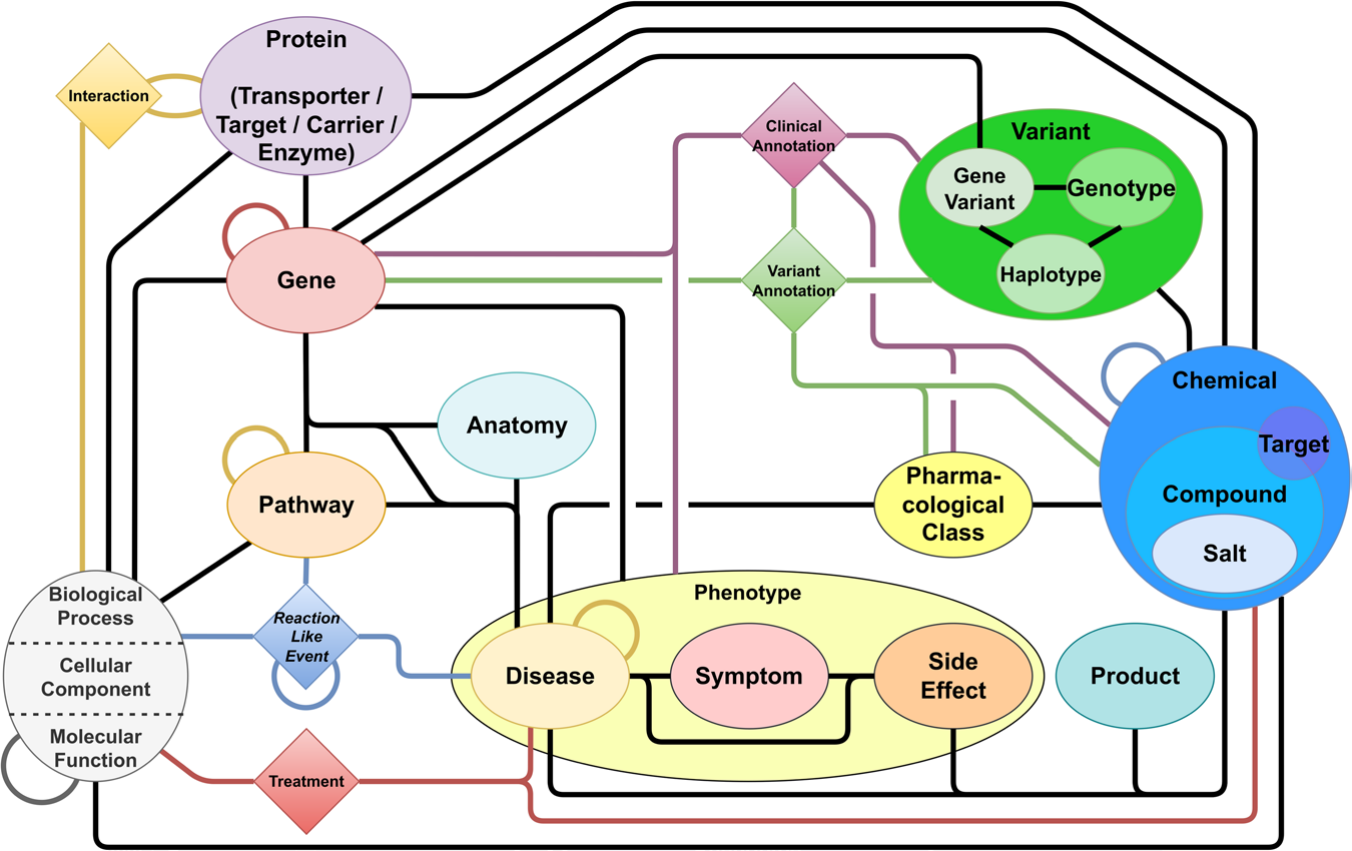
Schema of the PharMeBINet database. The different node labels are shown and how they are linked together. Multiple labels illustrate how entities are grouped together. Nodes in a diamond shape represent node edges that are used to combine connections between more than two nodes.

**Fig. 2:**
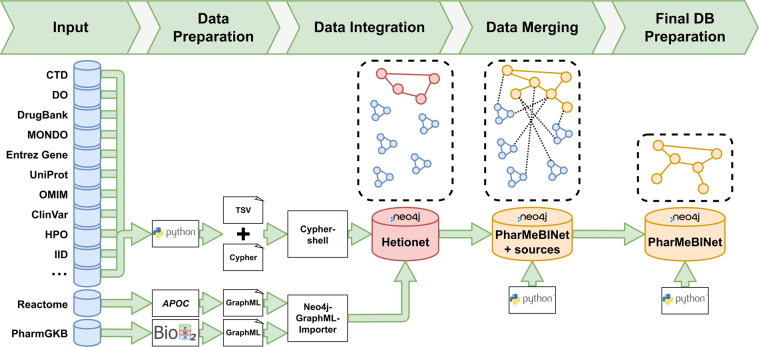
Construction of PharMeBINet in four steps. First, all data sources are prepared and exported to GraphML or TSV and Cypher files respectively. Second, the prepared files are integrated into Hetionet. Third, all data sources are merged or added to the Hetionet structure. Last, the final PharMeBINet database is created by removing all data source specific subgraphs, leaving only the merged graph.

Therefore, a heterogeneous pharmacological medical biochemical network (PharMeBINet) is constructed as a heterogeneous open-source Neo4j database. The schema of the database is visualized in Fig. 1. It combines the Hetionet database with 19 other data sources which are shown in Table 1. Hetionet was chosen as a starting point as it already combines 29 public resources, has a fitting license, and was successfully utilized in previous projects at our group. They are combined in the steps demonstrated in Fig. 2. PharMeBINet contains 66 node labels with 2,869,407 nodes and 208 edge types with a total number of 15,883,653 edges.

**Table 1:** List of data sources integrated into PharMeBINet with version, license, and reference.

Data Source	Version	License	Cite
Adverse Event Open Learning through Universal Standardization (AEOLUS)	2017-04-08	CC0 1.0	^42^
ClinVar	2022-05-05	NCBI license	^28^
Comparative Toxicogenomics Database (CTD)	2022-04	academic purposes	^3^
dbSNP	2021-05-25	NCBI license	^45^
Disease Ontology (DO)	2022-04-28	CC0 1.0	^34^
DrugBank	5.1.9	CC BY-NC 4.0	^2^
Entrez Gene	2022-05-11	NCBI license	^35^
Gene Ontology (GO)	2022-03-22	CC BY 4.0	^10,11^
Hetionet	1.0	CC0 1.0	^8^
Human Phenotype Ontology (HPO)	2022-04-14	custom	^32^
Integrated Interactions Database (IID)	2021-05	academic purposes	^36^
Mondo Disease Ontology (Mondo)	2022-05-02	CC BY 4.0	^33^
National Drug File-Reference Terminology (NDF-RT)	2018-02-05	UMLS license	^53^
Online Mendelian Inheritance in Man (OMIM)	2022-04-14	custom	^38^
Pathway Commons	12	multiple	^43^
Pharmacogenomics Knowledgebase (PharmGKB)	2022-05-05	CC BY-SA 4.0	^1^
Reactome	2022-03-31	CC BY 4.0	^40^
Side Effect Resource (SIDER)	4.1	CC BY-NC-SA 4.0	^41^
Universal Protein database (UniProt)	2022-01	CC BY 4.0	^37^
WikiPathways	2022-04-10	CC BY 3.0	^44^

## Methods

**Fig. 3:**
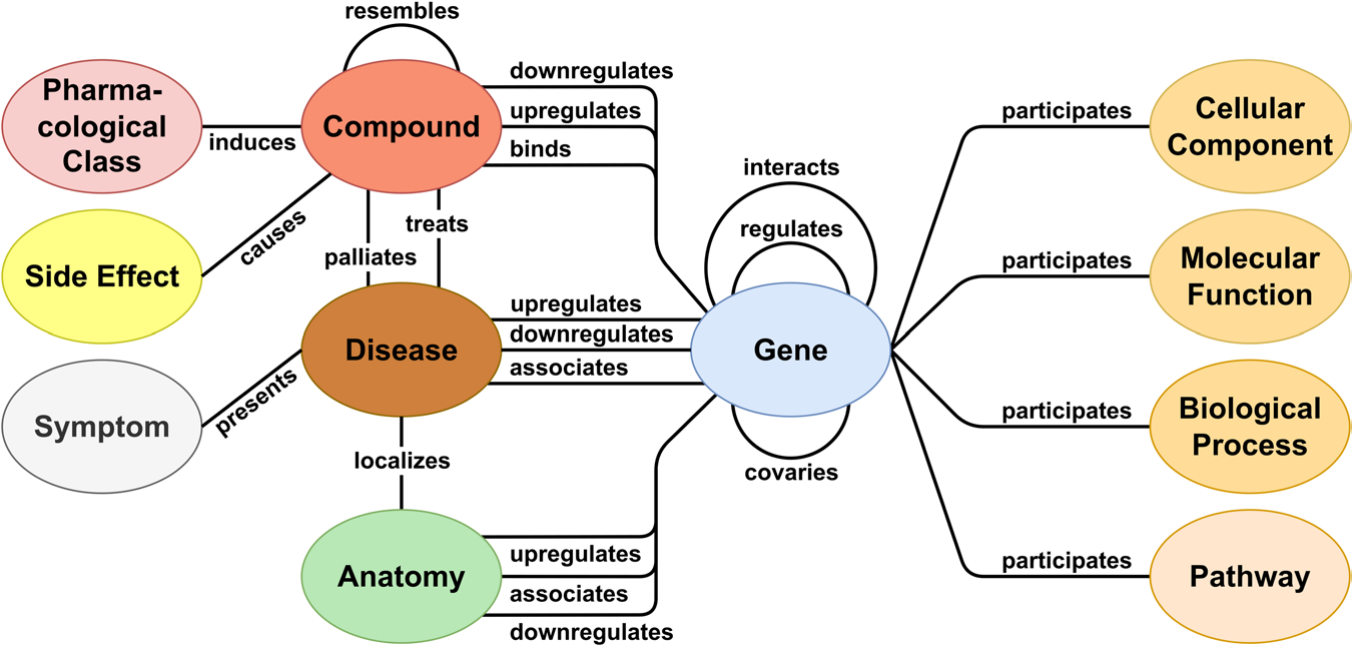
The schema of the Hetionet 1.0 database is visualized as ovals for the different node labels and connecting lines for the different edge types^8^.

The basis of PharmaMeBINet is Hetionet v1.0^8^, which is already a biomedical Neo4j database. The general structure representing the node labels and edge types of the Hetionet database is shown in Fig. 3. However, Hetionet lacks information for example on proteins and gene variants, has a low number of disease nodes (136), and edge types for the induction of diseases by drugs are missing. Therefore, more databases (Table 1) are considered and integrated into Neo4j.

PharMeBINet is constructed as a Neo4j V4.2.5 graph database in four distinct steps: data preparation, integration, merging, and final preparation as visualized in Fig. 2. The data preparation step solves the problem of heterogeneous data formats for different data sources. Each data source needs to be transformed into common file formats suitable for integration. This necessitates the development of parsers able to handle these transformations. The generated files from the data preparation step are then integrated into the Hetionet Neo4j database with all resulting subgraphs of the data sources still being disconnected. In the merging step, all data source subgraphs are queried for relevant information, processed, and merged or added to the Hetionet structure. This merging process is executed in a specific order of data sources depending on the source’s provided information and relevance. The final database preparation step constructs the public PharMeBINet database by removing all data source specific subgraphs leaving only the merged graph schema. Following, these steps are described in detail.

### Data source preparation

All preparation steps for the different data sources entail some form of file download, parsing, and export. The heterogeneous file formats required to be parsed are tab-separated values (TSV), comma-separated values (CSV), Extensible Markup Language (XML), Open Biomedical Ontologies (OBO), Spatial Data File (SDF), FASTA, and JavaScript Object Notation (JSON). As the Python 3 programming language is used for the whole process, parsers for JSON, XML, CSV, and TSV files are available without any additional libraries.

An SDF file represents a collection of objects with properties as key-value pairs. These objects are directly representable as rows of a TSV file for which a custom python script is developed.

OBO files represent entities and their relationships in ontological form. As multiple data sources use the OBO file format, a generic converter from OBO to TSV files is developed. This converter extracts all node information into a single TSV file with each row representing a node. All relationship types between nodes in the OBO file are extracted into separate TSV files. A Cypher file is generated as well with queries loading these TSV files and creating nodes and edges with their respective information.

FASTA files provide pairs of headers and sequences in plain text which may be amino-acid or nucleotide sequences. A simple parser is developed to load these header-sequence pairs which may then be further processed in the data sources.

All data sources are exported either to TSV or, in the case of Reactome and PharmGKB, GraphML files. This decision was made as many data sources either already provide all or some information in TSV format and the resulting files are easily inspected for correctness with text editors or software such as LibreOffice. Additionally, Cypher files are exported for each data source containing queries for loading the respective TSV files and the creation of nodes and edges in preparation for the subsequent integration step.

The Human Phenotype Ontology (HPO)^32^ provides the OBO file “hp.obo” as well as the annotations file “phenotype.hpoa” which is a TSV format. This OBO file contains information on symptoms and the annotation file provides relationship information between these symptoms and diseases. Both files are downloaded. Using the aforementioned OBO converter script, all information from the “hp.obo” is transformed into TSV files. Distinct diseases, as well as all relationships are extracted from the annotation file and stored in separate TSV files. The Cypher file prepared by the OBO converter is appended with queries to add the disease and relationship information from the annotation TSV files.

Mondo Disease Ontology (Mondo)^33^ is an ontology of diseases already merging several disease resources. The OBO file “mondo.obo” is downloaded and processed using the OBO converter into TSV and a Cypher file.

Disease Ontology (DO)^34^, as the name already states, is another ontology of diseases that provides the OBO file “doid.obo”. Like Mondo, the OBO file is downloaded and converted to TSV and a Cypher file.

From the National Center for Biotechnology Information (NCBI) the Entrez genes^35^ are downloaded using the available *Homo sapiens* file “Homo_sapiens.gene_info.gz” in compressed TSV format. However, the file also contains genes of *Homo sapiens subsp.’Denidova’* and *Homo sapiens neanderthalensis*. The preparation script filters them out by excluding all genes with a different NCBI taxonomy id than 9606. All remaining gene information is written to a TSV file without modification. A Cypher file is generated with queries for loading the TSV file and creating gene nodes with all information as properties.

Gene Ontology (GO)^10,11^ is a hierarchy of gene-associated terms for biological processes, molecular functions, and cellular components. The OBO ontology file “go-basic.obo” is downloaded and converted to TSV and Cypher files. Additionally, GO provides different annotation files for species and molecular entities associating GO terms with these molecular entities such as proteins or RNA. All human annotation files “goa_human.gaf.gz”, “goa_human_complex.gaf.gz”, “goa_human_isoform.gaf.gz”, and “goa_human_rna.gaf.gz” are downloaded. The GAF file format is a TSV format with header lines and can be processed just like TSV after skipping the header lines. Annotations are processed by translating their evidence abbreviation to a longer textual form and writing distinct molecular entities into separate TSV files per node label as well as TSV files for the different relationship types. The generated Cypher file is appended with queries for integrating the annotation nodes and edges.

From the Integrated Interactions Database (IID)^36^ the file “human_annotated_PPIs.txt.gz” is downloaded which only contains human protein interactions in compressed TSV format. Information on individual proteins is extracted from the file, deduplicated, and stored in a TSV file. Interaction relationship information is extracted and stored in another TSV file. A Cypher file is generated loading these TSV files and creating nodes and edges.

Protein data are extracted from the Universal Protein database (UniProt)^37^. Swiss-Prot is chosen as a UniProt subset as it contains only manually reviewed information. The XML file “uniprot_sprot_human.xml.gz” is downloaded containing human protein, disease, protein-disease relationship, and protein interaction information. Separate TSV files are generated for the entities protein, disease, evidence, and keyword with deduplicated information from the XML file. Relationships between protein and protein, disease, keyword, and evidence are extracted as well and written to separate TSV files. A Cypher file is generated with queries for integrating these TSV files.

For parsing Online Mendelian Inheritance in Man (OMIM)^38^ the four files in TSV format “mimTitles.txt”, “mim2gene.txt”, “genemap2.txt”, and “morbidmap.txt” are downloaded for which an API key is required but freely requestable. The “mim2gene.txt” contains basic information such as entity type and xrefs for each OMIM id. Similarly, the “mimTitles.txt” provides names, synonyms, and symbols. The other two files contain information about the relationships between genes and phenotypes in OMIM. Based on the OMIM ids the node information is combined, the same goes for OMIM pairs for the edges. All node and relationship information is written into TSV files per label. A Cypher file is generated with queries for loading these TSV files and generating nodes and edges from the information. Additionally, queries are generated to remove all gene and phenotype nodes without any relationships.

CTD provides information on many entity types such as chemicals, pathways, diseases, and more as well as their relationships^3^. All information is already available in TSV format and downloaded. For a full list of filenames see Supplementary Table 3. To ensure information of higher quality, relationships between chemical and disease as well as gene and disease are filtered out if the field “DirectEvidence” is empty. Cypher queries are generated for all entities and relationships to be loaded and created from the TSV files. Additionally, queries are generated to remove all gene, disease, pathway, and GO nodes without any relationships. Disjoint chemicals are not removed as they are useful for later mapping.

PharmGKB is focused on pharmacogenomic information like the effect of gene variants on drug-disease interactions^1^. The data integration tool BioDWH2 v0.4.0^39^ provides a suitable data source module for PharmGKB. It is used to generate a GraphML file from PharmGKB data representing a graph in XML format which is directly able to be integrated.

The pathway-focused resource Reactome^40^ is already available as the Neo4j database file “reactome.graphdb.tgz”. However, a Neo4j database cannot directly be transferred into another Neo4j database. Therefore, the Reactome graph is exported using Awesome Procedures on Cypher (APOC) version 4.2.0.5 (https://github.com/neo4j-contrib/neo4j-apoc-procedures) into a GraphML file for subsequent re-import.

Side Effect Resource (SIDER)^41^ provides drug-side effect and drug-indication information extracted from drug labels using text-mining. The files “meddra_all_se.tsv”, “meddra_all_label_indications.tsv”, “meddra_all_indications.tsv”, and “meddra_freq.tsv” are downloaded and further processed. Separate TSV files are generated for distinct drugs and side effects as well as for causes and indicates relationships. Relationship information is aggregated from the different source files. A Cypher file is generated with queries for node and edge creation from the TSV files.

Adverse Event Open Learning through Universal Standardization (AEOLUS)^42^ is a resource on adverse drug events generated from FAERS data. The data archive is downloaded from the publications dataset and the TSV files “standard_case_drug.tsv”, “standard_case_indication.tsv”, “concept.tsv”, “standard_drug_outcome_contingency_table.tsv”, and “standard_drug_outcome_statistics.tsv” are extracted. Separate TSV files are generated for distinct drugs and outcomes as well as for causes and indicates relationships. The causes edges have the combined information of the contingency table and statistics for each drug-outcome pair. A Cypher file is generated with queries for node and edge creation from the created TSV files.

Also integrated is ClinVar which aggregates information about genomic variation and its relationship to human health^28^. From the ClinVar FTP the XML files “ClinVarVariationRelease_00-latest.xml” and “ClinVarFullRelease_00-latest.xml” are downloaded. Additional use of the variation release is done as, at the time of implementation, not all variants are found in the full release file but are visible on the website on available in the variation release. The variation release contains information about genotypes, haplotypes, and gene variants and their relationships. Similarly, the full release provides the same information with the addition of information for different trait sets like diseases or drug responses. The genotype, haplotype, and gene variant information of both XML files are combined and written into separate TSV files as well as all the other types of entities in the full release. All available relationship types are combined and written to separate TSV files as well. Additionally, two Cypher files are generated. One for loading and integrating the node TSV files and the other for the edge TSV files.

Hetionet parses Pathway Commons^43^ and WikiPathways^44^ for pathway information and gene relationships. Similarly, the two GMT files “PathwayCommons12.All.hgnc.gmt.gz” and “wikipathways-20220410-gmt-Homo_sapiens.gmt” are downloaded from both data sources. These files each provide pathway names, descriptions, and associated sets of genes. This data source preparation step is so far different as it uses the output of the previously prepared Entrez Gene data source. Like in Hetionet, Entrez is used to filter out pathway-gene associations which are not present anymore in the current version of Entrez or which are not protein-coding. Pathway Commons provides species taxonomy ids for pathways which are filtered for human pathways only. Additionally, Pathway Commons combines pathways from multiple data sources with varying licenses. Only pathways from WikiPathways, Reactome, Panther, NetPath, and PathBank are retained as the licenses are suitable for PharMeBINet. As the WikiPathways file is already a human subset, no species filtering is required. Pathway and associated gene set information is combined from both data sources and stored in a TSV file. A Cypher file is generated with queries for loading and integrating this TSV file.

DrugBank^2^ is a comprehensive database focused on drugs and their relationship to targets, diseases, and more. Hetionet integrates only a tiny subset of information from the DrugBank XML file. For PharMeBINet not only all information from the DrugBank XML file “drugbank_all_full_database.xml.zip” is used but also all other files available for download. For a complete list of files see Supplementary Table 3. Additionally, DrugBank categories are extracted from the website as category identifiers are not provided in the XML file. The full DrugBank XML is processed and entities as well as relationships are extracted. After extraction from the XML file, this data must be combined with information from additional DrugBank files about structures, metabolites, external links, protein identifiers, target sequences, and drug sequences as they provide some additional data not present or differing in the XML file. For drug and metabolite structures the aforementioned custom SDF parser is executed on the SDF files converting them to TSV files for further processing. Sequence files are processed using the FASTA parser to be combined with the other file’s information. Protein information with UniProt identifiers in DrugBank is filtered using the output of the UniProt data source preparation step. All proteins in DrugBank which are not available in the UniProt list of identifiers and additional identifiers are removed to match the newest UniProt version and simplify subsequent mapping steps. TSV files are generated each for drugs, targets, carriers, transporters, enzymes, single-nucleotide polymorphisms (SNPs), pathways, salts, products, metabolites, and pharmacological classes with all provided information. Relationships between these entities are written to separate TSV files as well. A Cypher file for nodes and one for relationships is generated with queries for loading and integrating the generated TSV files.

The Single Nucleotide Polymorphism Database (dbSNP)^45^ is different from the other data sources because of its data file sizes. Therefore, only the dbSNP entries of the other data sources are extracted from the dbSNP API. This is executed after most mapping and merging steps are executed and described later.

### Data source integration

As mentioned before, each data source preparation step generates TSV as well as Cypher files for data integration. These Cypher files are executed one after the other using the cypher-shell command line tool bundled with Neo4j. The queries are executed on a running instance of the Hetionet Neo4j database.

The exceptions Reactome and PharmGKB are prepared as GraphML files. As the APOC plugin has issues with importing large GraphML files, the Neo4j-GraphML-Importer tool version 1.1.5 (https://github.com/BioDWH2/Neo4j-GraphML-Importer) is used to import both GraphML files into the Neo4j database. This has the advantage as the importer tool is able to generate node indices as well as rename node and edge labels from the GraphML files to match the used naming scheme for all data sources.

Afterwards, all data sources including Hetionet are available in one large Neo4j database but still disconnected from each other.

### Data source mapping and merging

The next step is the merging of all data source information as visualized in Fig. 2. For this, the data sources integrated in the previous step are mapped and merged with the existing Hetionet structure. Mapping entities from data sources means for example that a node *Aspirin* from DrugBank must be mapped to the Hetionet Compound node *Aspirin*. Mapping each data source is so far challenging as different entities require different mapping strategies^29,31^. For example, mapping via names is not feasible for all cases as spelling may vary across databases or synonyms as well as different languages may be used. If there are chemicals/drugs containing information on their structure these are more appropriate for the mapping process^46^. Another method is using external identifiers, which are cross-references to other sources for a certain entity^46^. It is possible to use external databases such as Unified Medical Language System (UMLS) (version 2021AB) and RxNorm (version 2021-11-12) which are among others used for the mappings of PharMeBINet. The decision of which mapping strategy is most suitable is adjusted according to the information provided by each data source.

This merging process is executed in a specific order as detailed below and listed in Supplementary Table 1 for all new and already existing labels from Hetionet. All mapping methods between entities are listed in Supplementary Table 2 as well. Following, all data source specific steps are described in detail and in the aforementioned order. The Hetionet naming scheme for edge types is continued throughout the mapping process. The relationship name is followed by an underscore, the uppercase abbreviation of the source node label, the lowercase abbreviation of the relationship name, and the uppercase abbreviation of the target node label.

PharMeBINet introduces a new hierarchy of the labels “Chemical” ⊃ “Compound” ⊃ “Salt”. To better fit this new hierarchy existing edges of “Compound” nodes are renamed to expose the “Chemical” label as follows:

- “CAUSES_CcSE” → “CAUSES_CHcSE”
- “PALLIATES_CpD” → “PALLIATES_CHpD”
- “TREATS_CtD” → “TREATS_CHtD”
- “BINDS_CbG” → “BINDS_CHbG”
- “DOWNREGULATES_CdG” → “DOWNREGULATES_CHdG”

Nodes with the label “Disease” are already present in Hetionet originating from DO. As Mondo includes DO together with other data sources all existing “Disease” nodes are replaced with matching Mondo information or removed if not present in Mondo. New “Disease” nodes are created from Mondo where no matching nodes exist. As Hetionet contains multiple relationships for “Disease” nodes from different data sources, those edges are preserved for the replaced nodes. Additionally, the new edge type “IS_A_DiaD” is introduced from Mondo. The grouping label “Phenotype” is added to “Disease” nodes as well.

As Mondo already merges the information from multiple data sources DO is mapped to Mondo using DO identifier, OMIM identifier, and name mapping and merged into the nodes to retain all information DO provides in combination with Mondo information. For example, DO provides definition texts for more diseases than Mondo. DO provides additional “IS_A_DiaD” relationships not present in Mondo which are added as well.

Nodes with the label “Gene” in Hetionet originate from Entrez Gene. To update the genes with the latest information all “Gene” nodes are replaced if still present in Entrez or otherwise removed and new ones are added. This is done analogous to the “Disease” nodes to preserve existing edges. No relationships are added or updated.

OMIM provides information on diseases and genes. Therefore, existing “Gene” nodes are matched using the Entrez gene identifier and information updated. Nodes with the label “Disease” are either matched using the OMIM identifier or created as a more general “Phenotype” node if matching fails. Associations between genes and diseases are added from OMIM as “ASSOCIATES_PaG” edges. Existing “ASSOCIATES_DaG” edges are matched and updated where possible and added otherwise.

Pathway Commons and WikiPathways are already integrated in Hetionet. To update the information all previous “Pathway” nodes and relationships are removed and new “Pathway” nodes added. “Gene” nodes are matched using the gene symbols in order to add “PARTICIPATES_IN_GpiPW” edges between genes and pathways.

For adding protein information new nodes with the label “Protein” are created from UniProt. Protein-protein interaction relationships in UniProt could be added as edges between two protein nodes. However, as other data sources introduce interactions with an additional relationship to GO terms they are added as an “Interaction” node and “INTERACTS_IiP” and “INTERACTS_PiI” edges respectively between the interaction and proteins. New “PRODUCES_GpP” edges are inferred and created from UniProt information of which “Gene” node produces which “Protein” node. Finally, the gene-disease relationship edge “ASSOCIATES_DaG” is either merged and updated or created. For this, “Gene” nodes are matched using the Entrez gene identifier, gene symbol, and name mapping. “Disease” nodes are matched using the OMIM identifier.

GO terms are already integrated in Hetionet. To update the information all previous “BiologicalProcess”, “CellularComponent”, and “MolecularFunction” nodes are removed and new ones created from the latest version of GO^10,11^. Additionally, Hetionet only provided “PARTICIPATES” edges between genes and GO terms although GO annotations provide a richer set of relationships. Previous GO term edges are removed and the GO term hierarchy added as “IS_A_BPiaBP”, IS_A_CCiaCC”, and “IS_A_MFiaMF” edges as well as all edges between gene or protein and GO terms. For mapping the gene products to “Gene” nodes, the gene symbol and gene name of the proteins are considered. “Protein” nodes are matched using the UniProt identifier. The full list of relationships is available in Supplementary Table 1.

IID^36^ provides more protein-protein interaction relationships for which “Protein” nodes are matched using UniProt identifiers and “Interaction” nodes as well as “INTERACTS_IiP” and “INTERACTS_PiI” edges are added. Additionally, interactions in IID provide a reference to cellular component GO terms for which “CellularComponent” nodes are matched using name mapping. “MIGHT_SUBCELLULAR_LOCATES_ImslCC” edges are introduced between “Interaction” and GO term nodes.

Reactome primarily provides pathway information for which existing “Pathway” nodes are either matched using Reactome identifiers and name mapping and updated or newly created. Relationships between pathways are added as edges with labels:

- “PRECEDING_REACTION_PWprPW”
- “NORMAL_PATHWAY_PWnpPW”
- “OCCURS_IN_PWoiPW”
- “HAS_ENCAPSULATED_EVENT_PWheePW”

Pathways in Reactome are composed of reaction-like events which provide relationships to multiple entities and are therefore added as edge nodes. Only human reaction-like events with at least one PubMed identifier are considered. Labels for these events are “Reaction”, “FailedReaction”, “BlackBoxEvent”, “Polymerisation”, and “Depolymerisation”. Additionally, the grouping label “ReactionLikeEvent” is added to these nodes. Relationship edges between reaction-like events and pathways are added with labels:

- “IS_NORMAL_REACTION_RLEinrRLE”
- “PRECEDING_REACTION_RLEprRLE”
- “HAS_EFFECT_ON_RLEheoRLE”
- “REVERSE_REACTION_RLErrRLE”
- “PRECEDING_REACTION_PWprRLE”
- “PRECEDING_REACTION_RLEprPW”
- “PARTICIPATES_IN_PWpiRLE”

Reactome also provides relationships from pathways and reaction-like events to diseases and GO terms. Nodes with the labels “BiologicalProcess”, CellularComponent”, and “MolecularFunction” are matched using GO identifiers and edges created with the labels:

- “IN_COMPARTMENT_RLEicCC”
- “OCCURS_IN_PWoiBP”
- “OCCURS_IN_RLEoiBP”
- “IN_COMPARTMENT_PWicCC”

“Disease” nodes are matched using DO identifier and name mapping and edges are created with the labels “LEADS_TO_DISEASE_RLEltdD” and “LEADS_TO_PWltD”. Treatment information from Reactome will be added at a later stage after chemicals have been merged.

To introduce variant information for a better understanding of molecular mechanisms ClinVar is added. As no variants were previously included they are directly added from ClinVar. Depending on the details provided, one of the labels “Haplotype”, “Genotype”, or “GeneVariant” is used to create new nodes with the additional grouping label “Variant”. Nodes with the label “Gene” are matched using Entrez gene identifier for introducing relationship edges with the labels “HAS_GhV”, “HAS_HhGV”, “HAS_GThH”, and “HAS_GThGV”.

Hetionet already provides “Compound” nodes integrated from DrugBank drugs with relationship edges from multiple sources. To update the information matching “Compound” nodes by their DrugBank identifier are retained and updated to preserve existing edges. Compounds not present in DrugBank anymore are removed. New compounds in DrugBank are created as “Compound” nodes. Salts and drug products from DrugBank are created as “Salt” and “Product” nodes. All “Salt” nodes also receive the broader “Compound” label. Salts are pharmaceutical salts of drugs that can increase their chemical stability and facilitate the manipulation of the agent’s pharmacokinetic profile. Drug products are added for additional information on applied drugs such as dosages. “GeneVariant” nodes are matched using dbSNP identifier or newly created from DrugBank adverse drug reaction information. Relationship edges are created with the labels “HAS_ChPR”, “COMBINATION_CAUSES_ADR_VccaCH”, “PART_OF_CpoSA”, and “INTERACTS_CiC”. The new “INTERACTS_CiC” edge representing drug-drug interactions is especially important for finding potential ADRs. Hetionet already provides a calculated “RESEMBLES_CrC” relationship between compound chemical structures. As DrugBank is updated these edges are removed and new ones calculated using multiple fingerprint metrics of RDKit (https://rdkit.org) and Open Babel Pybel^47,48^ and similarity measures such as Tanimoto or Dice coefficient. To retain only significant similarities a threshold of 0.75 is applied reducing the number of created edges.

HPO provides information on diseases and symptoms. Existing “Disease” nodes are matched using OMIM identifier, ORPHA identifier, UMLS identifier, and name mapping and updated with HPO information. No new “Disease” nodes are created as Mondo already provides a robust set of diseases. HPO nodes that are part of the phenotypic abnormality hierarchy are matched and updated or newly created as “Symptom” nodes. The mapping to symptoms uses Medical Subject Headings (MeSH) identifier, UMLS identifier, and name mapping. In contrast to diseases, symptom information in Hetionet originates from MeSH terms representing only a sparse subset of all symptoms. The grouping label “Phenotype” is added to “Symptom” nodes as well. Edges with the label “PRESENTS_DpS” already exist in Hetionet and are therefore either matched and updated or newly created from HPO.

CTD chemicals are mapped to “Compound” nodes with multiple mapping methods: Chemical Abstracts Service (CAS)-number, UMLS identifier, RxNorm identifier, UNII identifier, International Chemical Identifier Key (InChIKey), MeSH identifier, and name mapping. If a node is matched properties are updated, otherwise new nodes with the label “Chemical” are created. All nodes with the label “Compound” which already includes “Salt” nodes are assigned the additional grouping label “Chemical”. CTD genes are matched and updated to “Gene” nodes using Entrez gene identifier. Relationship edges are matched and updated or newly created between chemicals and genes with the labels “BINDS_CHbG” and “DOWNREGULATES_CHdG”. New edge labels between chemicals and genes are:

- “BINDS_GbCH”
- “ASSOCIATES_CHaG”, “ASSOCIATES_GaCH”
- “UPREGULATES_GuCH”, “UPREGULATES_CHuG”
- “DOWNREGULATES_GdCH”
- “AFFECTS_DEGENERATION_GadCH”
- “DECREASES_DEGENERATION_CHddG”
- “INCREASES_DEGENERATION_CHidG”, “INCREASES_DEGENERATION_GidCH”
- “IS_ACTIVE_IN_METABOLISM_CHiaimG”, “IS_ACTIVE_IN_METABOLISM_GiaimCH”
- “IS_ACTIVE_ON_CELLULAR_LEVEL_CHiaoclG”, “IS_ACTIVE_ON_CELLULAR_LEVEL_GiaoclCH”
- “IS_ACTIVE_ON_DNA_OR_RNA_LEVEL_CHiaodorlG”
- “IS_ACTIVE_ON_POLYPEPTIDE_LEVEL_CHiaoplG”, “IS_ACTIVE_ON_POLYPEPTIDE_LEVEL_GiaoplCH”

GO terms in CTD are matched with “BiologicalProcess”, “CellularComponent”, and “MolecularFunction” nodes using GO identifier and updated. Relationships between chemicals and GO terms are added as edges with labels:

- “ASSOCIATES_CHaBP”, “ASSOCIATES_CHaCC”, “ASSOCIATES_CHaMF”
- “DECREASES_CHdBP”, “DECREASES_CHdCC”, “DECREASES_CHdMF”
- “INCREASES_CHiBP”, “INCREASES_CHiCC”, “INCREASES_CHiMF”

CTD pathways are matched to “Pathway” nodes using Reactome identifier and name mapping and the relationship edge “PARTICIPATES_IN_GpiPW” is either matched and updated or newly created between genes and pathways. “Disease” nodes are matched from CTD using OMIM identifier, MESH identifier, DO identifier, and name mapping. Edges with the label “TREATS_CHtD” are either matched and updated or newly created between chemicals and diseases. New edge labels “INDUCES_CHiD” and “ASSOCIATES_DaG” are created between diseases and chemicals or genes respectively. Relationships in CTD between chemicals and genes provide a flag whether a gene or protein is the targeted entity. In case of protein relationships “Protein” nodes are matched based on the previous “Gene” node mapping. Relationship edges between chemicals and proteins are created with the labels:

- “BINDS_CHbP”, “BINDS_PbCH”
- “ASSOCIATES_CHaP”, “ASSOCIATES_PaCH”
- “UPREGULATES_CHuP”, “UPREGULATES_PuCH”
- “DOWNREGULATES_CHdP”, “DOWNREGULATES_PdCH”
- “AFFECTS_DEGENERATION_CHadP”, “AFFECTS_DEGENERATION_PadCH”
- “DECREASES_DEGENERATION_CHddP”, “DECREASES_DEGENERATION_PddCH”
- “INCREASES_DEGENERATION_CHidP”, “INCREASES_DEGENERATION_PidCH”
- “IS_ACTIVE_IN_METABOLISM_CHiaimP”, “IS_ACTIVE_IN_METABOLISM_PiaimCH”
- “IS_ACTIVE_ON_CELLULAR_LEVEL_CHiaoclP”, “IS_ACTIVE_ON_CELLULAR_LEVEL_PiaoclCH”
- “IS_ACTIVE_ON_DNA_OR_RNA_LEVEL_CHiaodorlP”, “IS_ACTIVE_ON_DNA_OR_RNA_LEVEL_PiaodorlCH”
- “IS_ACTIVE_ON_POLYPEPTIDE_LEVEL_CHiaoplP”, “IS_ACTIVE_ON_POLYPEPTIDE_LEVEL_PiaoplCH”

All additions of relationship edges from CTD are filtered for human relationships with publication references.

National Drug File-Reference Terminology (NDF-RT) provides information on chemicals and diseases. Hetionet already provides pharmacological classes from the U.S. Food and Drug Administration (FDA) via DrugCentral with NDF-RT identifiers. To update this information all “PharmacologicalClass” nodes and “INCLUDES_CiPC” edges are removed and new “PharmacologicalClass” nodes are created from NDF-RT mechanism of action, physiologic effect, pharmacokinetics, and therapeutic category. The hierarchy of classes is represented by new edges with the label “INCLUDES_PCiPC”. NDF-RT drugs and ingredients are mapped to “Chemical” nodes using RxNorm identifier, UMLS identifier, InChIKey, UNII identifier, and name mapping. UMLS identifier, MeSH identifier, and name mapping are used to match and update “Disease” nodes from NDF-RT diseases. Relationships between chemicals, pharmacological classes, and diseases are newly created with the labels:

- “CONTRAINDICATES_CHcCH”
- “CONTRAINDICATES_CHcPC”
- “CONTRAINDICATES_CHcD”
- “TREATS_PCtD”
- “HAS_ACTIVE_METABOLITE_CHhamCH”
- “HAS_CHEMICAL_STRUCTURE_CHhcsCH”
- “HAS_CHEMICAL_STRUCTURE_PChcsCH”
- “HAS_INGREDIENT_CHhiCH”
- “INCLUDES_PCiCH”
- “MAY_DIAGNOSES_CHmdD”
- “MAY_DIAGNOSES_PCmdD”
- “METABOLIZES_PCmCH”
- “PREVENTS_CHpD”
- “PREVENTS_PCpD”

Existing edges with labels “INDUCES_CHiD” and “TREATS_CHtD” are matched or newly created.

After chemicals are merged into PharMeBINet the remaining information from Reactome can be merged as well. Treatment information from Reactome connects chemicals with diseases and cellular components for which edge nodes are created with the label “Treatment”. “Disease” and “CellularComponent” nodes are matched as described before and “Chemical” nodes are matched using Chemical Entities of Biological Interest (ChEBI) identifier, KEGG identifier, PubChem identifier, International Chemical Identifier (InChI), International Union of Basic and Clinical Pharmacology (IUPHAR) identifier, and name mapping. Relationships between “Treatment” edge nodes and the respective nodes are created as edges with the labels “TREATS_CHtT”, “TREATS_TtD”, and “IS_LOCALIZED_IN_TiliCC”. As chemicals serve as inputs and outputs of Reactome reactions, edges are created between “ReactionLikeEvent” and “Chemical” nodes with labels “HAS_INPUT_RLEhiCH” and “HAS_OUTPUT_RLEhoCH”. Finally, proteins are also possible inputs of reaction-like events for which “Protein” nodes are matched using UniProt identifier and edges with the label “IS_INPUT_OF_PiioRLE” are created.

ClinVar additionally provides relationship information between chemicals and variants. “Chemical” nodes are matched using name mapping from ClinVar drugs and edges are created with the labels:

- “ASSOCIATES_TO_DOSAGE_VatdCH”
- “ASSOCIATES_TO_EFFICACY_DOSAGE_VatedCH”
- “ASSOCIATES_TO_EFFICACY_METABOLISM_PK_DOSAGE_VatempdCH”
- “ASSOCIATES_TO_EFFICACY_TOXICITY_ADR_DOSAGE_VatetadCH”
- “ASSOCIATES_TO_EFFICACY_VateCH”
- “ASSOCIATES_TO_METABOLISM_PK_DOSAGE_VatmpdCH”
- “ASSOCIATES_TO_METABOLISM_PK_TOXICITY_ADR_VatmptaCH”
- “ASSOCIATES_TO_METABOLISM_PK_VatmpCH”
- “ASSOCIATES_TO_TOXICITY_ADR_DOSAGE_VattadCH”
- “ASSOCIATES_TO_TOXICITY_ADR_EFFICACY_VattaeCH”
- “ASSOCIATES_TO_TOXICITY_ADR_VattaCH”
- “ASSOCIATES_TO_TOXICITY_VattCH”
- “ASSOCIATES_VaCH”

After “Chemical” and “PharmacologicalClass” nodes have been introduced the remaining information of DrugBank can be integrated. DrugBank provides the Anatomical Therapeutic Chemical (ATC) code hierarchy for which existing “PharmacologicalClass” nodes are matched using name mapping and updated or newly created. The only exception is the lowest ATC level which is omitted as it is already represented by compounds. “Chemical” and “PharmacologicalClass” nodes are matched to add relationships as edges with the labels:

- “ASSOCIATES_CHaCH”
- “BINDS_CHbCH”
- “DEGENERATES_CHdCH”
- “DOWNREGULATES_CHdCH”
- “IS_ACTIVE_IN_METABOLISM_CHiaimCH”
- “REGULATES_CHrCH”
- “IS_ACTIVE_ON_POLYPEPTIDE_LEVEL_CHiaoplCH”
- “BELONGS_TO_CHbtPC”
- “BELONGS_TO_PCbtPC”

Information on drug-target relationships is extracted from DrugBank as well. For this, “Protein” nodes are matched using UniProt identifiers and information updated as well as more specific labels “Target”, “Transporter”, “Carrier”, and “Enzyme” added. Edges with the following labels are matched and updated or newly created between proteins and chemicals:

- “UPREGULATES_CHuP”
- “DOWNREGULATES_CHdP”
- “IS_ACTIVE_IN_METABOLISM_CHiaimP”
- “ASSOCIATES_CHaP”
- “IS_ACTIVE_ON_CELLULAR_LEVEL_CHiaoclP”
- “IS_ACTIVE_ON_DNA_OR_RNA_LEVEL_CHiaodorlP”
- “BINDS_CHbP”
- “IS_ACTIVE_ON_POLYPEPTIDE_LEVEL_CHiaoplP”

New relationships are created between chemicals and proteins with edge labels:

- “DEGENERATES_CHdP”
- “INHIBITS_CHiP”
- “IS_ACTIVE_AS_ANTIBODY_CHiaaaP”
- “REGULATES_CHrP”

Side effect information in Hetionet originated from SIDER. To update the information all “SideEffect” nodes and “CAUSES_CHcSE” edges are removed and newly created from SIDER. For this existing “Chemical” nodes are matched using PubChem identifier, InChIKey, DrugBank identifier, ChEMBL identifier, STITCH identifier, and name mapping and updated from SIDER information. The grouping label “Phenotype” is added to “SideEffect” nodes as well.

AEOLUS provides information on adverse drug events (named outcome) which are either matched and updated or added as new “SideEffect” nodes. The relationship edges “MIGHT_CAUSES_CHmcSE” and “MIGHT_INDUCES_CHmiD” are added by matching existing “Chemical” and “Disease” nodes. Side effects and diseases are mapped with multiple methods like extracting UMLS identifiers from BioPortal (https://bioportal.bioontology.org) via Medical Dictionary for Regulatory Activities (MedDRA) identifier, and name mapping. RxNorm identifier, UNII identifier, InChIKey, MeSH identifier, and name mapping are used for mapping AEOLUS drugs to chemicals. AEOLUS drug-outcome relationships are filtered so that only edges with a frequency of at least 0.1% and which appear at least 100 times are considered. This should remove drug-outcome pairs which are likely uncorrelated.

The merging of PharmGKB is split into two steps. First, all entities of interest are added. Nodes with the labels “Chemical” and “PharmacologicalClass” are matched using InChI, PubChem identifier, RxNorm identifier, DrugBank identifier, NDF-RT identifier, MeSH identifier, UMLS identifier, and name mapping and information is updated from PharmGKB. “Gene” nodes are matched using Entrez gene identifier, gene symbol, and name mapping and updated. PharmGKB phenotypes are mapped to “Disease”, “Symptom”, and “SideEffect” nodes using UMLS identifier, SNOMED identifier, and name mapping. Phenotypes that could not be mapped are added as nodes with the label “Phenotype”. “Variant” nodes are matched from PharmGKB using dbSNP identifier and name mapping and updated. For variants that could not be matched new “GeneVariant” nodes are created. The merging of relationships is done after dbSNP has been merged as gene variants may be deleted in that step.

Some variants from DrugBank and PharmGKB can not be connected to ClinVar, but have a dbSNP identifier. As dbSNP is a very large resource, only information is extracted from the API where the rs-identifier (dbSNP identifier) is present in the other data sources. The results from the API are in JSON format. Matching “GeneVariant” nodes are updated with information from dbSNP. Nodes with the label “GeneVariant” and an rs-identifier that are not present anymore in the current dbSNP version are removed to provide a robust subset of gene variants in PharMeBINet. Edges with the label “COMBINATION_CAUSES_ADR_VccaCH” are removed as well if the “GeneVariant” node is removed. Edges with the label “HAS_GhV” are added if not already existent by matching “Gene” nodes for their respective gene variant using Entrez gene identifier.

In the last step, PharmGKB relationship edges are added. For this, nodes with the labels “Variant”, “Gene”, “Chemical”, “Phenotype”, and “PharmacologicalClass” are matched as described before. “HAS_GhV” edges are either matched or newly created between genes and variants. Complex relationships as provided by PharmGKB are created as edge nodes “ClinicalAnnotation” and “VariantAnnotation”. Only clinical annotations with an evidence level of 1, 2, or 3 and variant annotations marked as significant are considered. Edges between these annotation nodes and the respective entities are then created with the labels:

- “ASSOCIATES_CAaG”
- “ASSOCIATES_CAaV”
- “ASSOCIATES_CAaCH”
- “ASSOCIATES_CAaPT”
- “ASSOCIATES_CAaPC”
- “ASSOCIATES_VAaG”
- “ASSOCIATES_VAaV”
- “ASSOCIATES_VAaCH”
- “ASSOCIATES_VAaH”
- “ASSOCIATES_VAaPC”
- “HAS_EVIDENCE_CAheVA”

In the end, relationships are generated between “Phenotype”, “SideEffect”, “Symptom”, and “Disease” nodes based on UMLS identifiers and name mapping to connect nodes of equal meaning. Created edge labels are respectively “EQUAL_DeSE”, “EQUAL_DeS”, “EQUAL_PTeSE”, and “EQUAL_SeSE”. Furthermore, allele relationships between “Variant” nodes are generated as edges with the label “IS_ALLEL_OF_ViaoV” from dbSNP identifiers provided by ClinVar.

### Preparation of the final database

In the last step, a database version with only the mapped and merged PharMeBINet schema is generated as shown in Fig. 2. First, the whole database from the merge step is exported as a GraphML file using APOC. Then, all database indices are extracted with a python script and a new bash script is prepared for the integration of a GraphML file using the Neo4j-GraphML-Importer tool. Next, a python script goes through the GraphML file and only transfers the PharMeBINet nodes and edges into another GraphML file. The bash script is executed which in turn executes the Neo4j-GraphML-Importer with the newly generated GraphML file and the fitting indices. The result is a clean Neo4j database without the data source subgraphs.

## Data Records

The dataset of PharMeBINet is available as a Neo4j database and GraphML download at https://pharmebi.net/#/download. As a permanent version record, the Neo4j database is deposited on Zenodo under 10.5281/zenodo.6578218^49^. The data may also be browsed on the website. A view exists for each node in the graph, where the properties of the node and all relationships are available. All data sources with versions that are used for the construction are shown in Table 1.

## Technical Validation

For the data sources Entrez Gene, UniProt, IID, ClinVar, and WikiPathways only the human data files are used to ensure that only human information is present for “Gene”, “Protein”, “Pathway”, and “Variant”. Entrez genes are filtered, even though it was a human file because it also contains some *Homo sapiens* sub-species information. The SwissProt subset of UniProt is used as it only provides manually reviewed protein information. Protein interactions from IID are filtered to only include those with experimental evidence and no predicted interactions. For CTD only the edges with literature references are integrated. In Reactome, only human reaction-like event nodes with at least one PubMed reference are retained. Similarly, only UniProt gene-disease edges with at least one PubMed reference are retained. Clinical annotations from PharmGKB are filtered to only include those with an evidence level of 1, 2, or 3. Additionally, variant annotations from PharmGKB are filtered for the significance property being equal to “yes”. Compound-protein edges from DrugBank are only merged into PharMeBINet if they include reference information.

The different mapping methods are checked semi-manually. This means for each mapping method mapped nodes are checked to have the same name and for those where this is not the case, multiple cases are checked manually to decide which mapping methods are suitable and which are not. Also, all data for “Variant”, “Gene”, “Protein”, “SideEffect”, “Symptom”, and “Phenotype” are checked that they are human data.

A python script is created to validate the naming scheme of relationship types which is adhered to for all edges resulting in a consistent final database. Besides, a validation is implemented that verifies for all data source names in the resource property of a node or edge the existence of a corresponding tag property.

## Usage Notes

PharMeBINet is downloadable as a Neo4j database or GraphML file on the web application (available at https://pharmebi.net) and is publicly available for researchers. This allows other researchers to use this database for their own research as machine learning as it was down with Hetionet 1.0^8^. Also, the web application allows the retrieval of information from the database and performs small analyses.

**Fig. 4:**
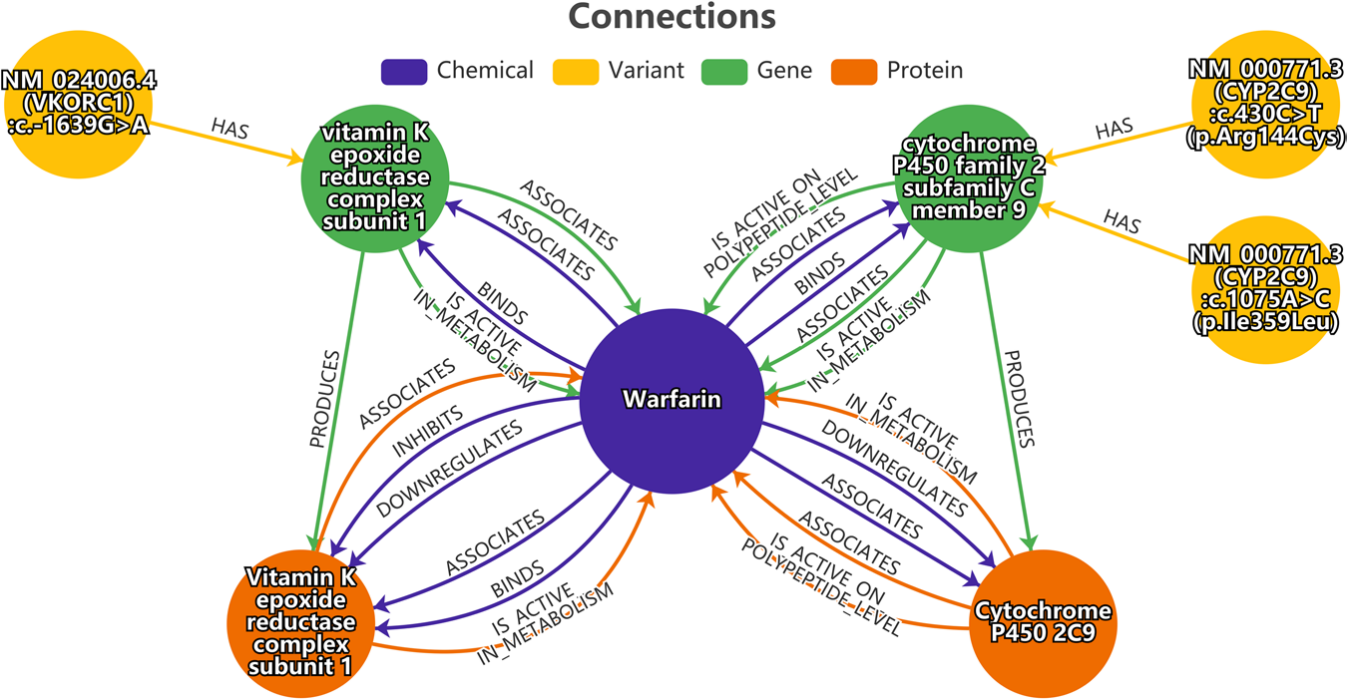
The resulted graph of the gene variants CYP2C9*2 (NM_000771.3(CYP2C9):c.430 C > T (p.Arg144Cys)), CYP2C9*3 (NM_000771.3(CYP2C9):c.1075 A > C (p.Ile359Leu)) and *VKORC1-1639 G > A* (NM_024006.4(VKORC1):c.-1639G > A), and the drug warfarin for the check of possible ADR caused by gene variant. All variants are connected via genes to chemicals and also the path from variants to genes to proteins to chemicals. The blue one is a chemical, the gene variant is yellow, the gene is green and the protein is orange.

One application is the analysis of possible existing connections between gene variants and drugs. As described before, genetic variants can cause potential ADRs. Therefore, the connection between a drug with its wild-type gene and the considered protein is analyzed. If this is the case, the connection between the drug, the gene/protein, and the variant is shown. It additionally shows a possibility for ADR because changes in gene or protein structure can modify the properties of the protein and cause different effects of this drug. An example is the selection of the drug warfarin and the gene variants of the gene cytochrome P-450 family 2, subfamily C, polypeptide 9 (*CYP2C9*) and the gene coding for vitamin K epoxide reductase complex subunit 1 (*VKORC1*). The gene product of *CYP2C9* is an essential enzyme, which is expressed in the liver for carbohydrate-, fat- and protein metabolism. The gene *VKORC1* encodes an enzyme that changes vitamin K to its active form. The selected gene variants of the *CYP2C9* are CYP2C9*2 (change in codon 144: arginine to cysteine) and CYP2C9*3 (change in codon 359:isoleucine to leucine) cause a reduced activity of the enzyme coding by *CYP2C9*^50^. For gene *VKORC1* the variant with an exchange of the nucleotide G to A in position 1,639 is called *VKORC1-1639 G > A*. This change is located in the gene *VKORC1* promoter region. This causes a lower promoter activity which results in a reduced number of proteins^50,51^. Figure 4 shows that for each of the gene variants a connection to warfarin over gene and protein exists. In conclusion, this combination has a high chance for ADR.

In the article of Mansouri *et al*.^50^ it is shown that the combination of these three gene variants in a person results in a different impact of warfarin than in people without these variants. For this reason, people having these gene variants need a lower dose of warfarin than normal people to get the same pharmacological effect. So, people without knowing their gene variants would take an overdose of warfarin and increase the risk of bleeding.

## Supplementary information

Supplementary Table 1

Supplementary Table 2

Supplementary Table 3

## Data Availability

The script to generate PharMeBINet is available from Zenodo^52^ and from GitHub https://github.com/ckoenigs/PharMeBINet. The repository contains python (3.8.5) programs and bash (4.4.20) scripts to execute all python programs, download databases, and execute the Neo4j Cypher tool. The website is available at https://pharmebi.net. The download of PharMeBINet is provided on the website.
